# Effects of Medicare Drug Subsidies on Adherence for Diabetics: Evidence From a Regression Discontinuity Design

**DOI:** 10.1093/geroni/igaa057.896

**Published:** 2020-12-16

**Authors:** Alexandra Glynn, Inmaculada Hernandez, Eric Roberts

**Affiliations:** University of Pittsburgh, Pittsburgh, Pennsylvania, United States

## Abstract

Out-of-pocket prescription drug costs are rapidly rising, particularly for insulin, which is a life-saving drug used by 3.1 million diabetics on Medicare. High out-of-pocket costs place an accentuated financial strain on older adults with diabetes, many of whom have low incomes, and may impede medication adherence, leading to poor health outcomes. The Medicare Part D Low-Income Subsidy (LIS) program limits drug co-pays to under $8.50 per prescription and caps out-of-pocket drug costs for lowest-income recipients (<135% Federal Poverty Level, FPL), resulting in pronounced differences in out-of-pocket costs for those with marginally different incomes. Using detailed income data from the Health and Retirement Study linked to Medicare claims (2008-2016), we employed a regression discontinuity (RD) design to isolate the effects of differences in out-of-pocket costs at eligibility thresholds for the LIS. Diabetic beneficiaries whose income exceeded the LIS eligibility threshold had lower Part D spending (-$945/year, p=0.03, n=2,367) and adherence to oral antidiabetic drugs (-8%, p=0.02). We conducted secondary analyses at the eligibility threshold for Medicaid, as individuals whose income exceeds the eligibility limit for Medicaid (100% of FPL in most states) are significantly less likely to receive the LIS. Above the Medicaid eligibility threshold (n=2,295), annual spending on insulin was $395 lower (p=0.002) and proportion of insulin use was 6% lower (p=0.04). These results suggest low-income Medicare beneficiaries who are not shielded from out-of-pocket costs via the LIS are particularly sensitive to drug costs. Policy proposals to limit out-of-pocket costs could improve medication adherence to high-cost drugs for vulnerable beneficiaries.

